# Effects of olanzapine treatment on lipid profiles in patients with schizophrenia: a systematic review and meta-analysis

**DOI:** 10.1038/s41598-020-73983-4

**Published:** 2020-10-12

**Authors:** Rong Li, Yiqi Zhang, Wenqiang Zhu, Chen Ding, Wenjie Dai, Xin Su, Wen Dai, Jingmei Xiao, Zhenhua Xing, Xiansheng Huang

**Affiliations:** 1 Department of Stomatology, The Second Xiangya Hospital, Central South University, Changsha, Hunan China; 2 Department of Cardiovascular Medicine, The Second Xiangya Hospital, Central South University, Changsha, Hunan China; 3 Department of Epidemiology and Health Statistics, Xiangya School of Public Health, Central South University, Changsha, Hunan China; 4 Department of Mental Health, The Second Xiangya Hospital, Central South University, Changsha, Hunan China; 5 Department of Emergency Medicine, The Second Xiangya Hospital, Central South University, Changsha, Hunan China

**Keywords:** Cardiovascular diseases, Metabolic disorders, Psychiatric disorders

## Abstract

Olanzapine-induced dyslipidemia significantly increases the risk of cardiovascular disease in patients with schizophrenia. However, the clinical features of olanzapine-induced dyslipidemia remain hitherto unclear because of inconsistencies in the literature. This meta-analysis thus investigated the effects of olanzapine treatment on lipid profiles among patients with schizophrenia. Studies of the effects of olanzapine on lipids were obtained through the PubMed, Web of science, The Cochrane Library and Embase databases (up to January 1, 2020). Twenty-one studies and 1790 schizophrenia patients who received olanzapine therapy were included in our analysis. An olanzapine-induced increase was observed in plasma triglyceride (TG), total cholesterol (TC), and low-density lipoprotein cholesterol (LDL-C) levels in patients with schizophrenia (all *P* < 0.05). Moreover, the time points analyzed included the following: baseline, 4 weeks, 6 weeks, 8 weeks, 12 weeks, and ≥ 24 weeks (data of ≥ 24 weeks were integrated). The significant elevation of TG, TC, and LDL-C was observed in patients with schizophrenia already by 4 weeks of olanzapine therapy (all *P* < 0.05), with no obvious changes observed in high-density lipoprotein cholesterol (HDL-C) (*P* > 0.05). In conclusion, olanzapine-induced dyslipidemia, characterized by increased TG, TC, and LDL-C levels, was observed in patients with schizophrenia already by 4 weeks of olanzapine treatment.

## Introduction

Schizophrenia is a severe neuropsychiatric illness that threatens approximately 1% of the global population^1^. High morbidity and mortality caused by dyslipidemia concomitant with cardiovascular disease (CVD) have been documented among patients with schizophrenia^2,3^. It has been shown that olanzapine, a second-generation antipsychotic drug, is a major contributor to dyslipidemia among patients with schizophrenia^4–6^. However, the clinical features of olanzapine-induced dyslipidemia remain unclear because of inconsistent data among studies. Although some clinical randomized trials have reported increased serum triglyceride (TG) levels in schizophrenic patients on olanzapine therapy, the changes in total cholesterol (TC), high-density lipoprotein cholesterol (HDL-C), and low-density lipoprotein cholesterol (LDL-C) remain a matter of dispute^7–11^.

Dyslipidemia is a well-established risk factor in the pathogenesis of CVD, including coronary heart disease (CAD), and acute coronary syndrome^12^. In fact, it is thought to account for more than half of the worldwide cases of CAD. Importantly, high plasma TG and LDL-C levels and/or low serum HDL-C levels have been implicated in the pathophysiology of CVD^13^. Accordingly, understanding the characteristics of olanzapine-induced dyslipidemia is critical to the assessment and control of cardiovascular risk in schizophrenic patients. We therefore conducted a systematic review and meta-analysis of published literature to determine olanzapine-associated lipid profiles in patients with schizophrenia. In addition, we further analyzed the influence of clinical characteristics and treatment duration on olanzapine-induced dyslipidemia in patients with schizophrenia.

## Methods

### Search strategy and study identification

This systematic literature review and meta-analysis was strictly subject to the Preferred Reporting Items for Systematic Reviews and Meta-Analyses (PRISMA) guidelines (see PRISMA Checklist in Supplementary table S1)^14^. We performed a comprehensive literature search in the following electronic databases to obtain eligible studies published by January 1, 2020: PubMed, Web of science, The Cochrane Library and Embase. The search terms were [‘schizophrenia’] AND [‘olanzapine’] AND [‘dyslipidemias’ OR ‘triglycerides’ OR ‘Cholesterol’ OR ‘Cholesterol, LDL’ OR ‘Cholesterol, HDL’]. We also adopted Medical Subject Headings (MeSH) and related keywords in our analysis to increase the number of studies in our search. Moreover, a manual search of reference lists was undertaken for additional relevant articles. English was the only standard language that was applied in the search strategy. If relevant data of published studies were not available, we contacted the corresponding author(s) to obtain them via email.

### Study selection

The inclusion and exclusion criteria of the obtained reports are described in reference to population, interventions, outcomes, and study design as follows.

### Population

People with schizophrenia diagnosed according to the criteria outlined in the *Diagnostic and Statistical Manual of Mental Disorders—Fourth Edition* (DSM-IV), the *International Statistical Classification of Diseases and Related Health Problems—Tenth Revision* (ICD-10), or the *3rd edition of the Chinese Classification of Mental Disorders* (CCMD-3) (the diagnostic criteria commonly used in China). No limitations were defined by sex, race, or age. Women were excluded if they were planning to be pregnant or were undergoing pregnancy or lactation. Moreover, patients with chronic physical illness were also excluded, including those with atherosclerotic CVD, hepatic diseases, renal diseases, and diabetes.

### Interventions

Only studies that administered olanzapine alone were considered. Moreover, all of the included studies were required to have provided detailed descriptions of the durations of administration. The dosage of olanzapine was further required to have been within the maximum dose range.

### Outcomes

The mean and standard deviation (SD) of the plasma levels of TG, TC, LDL-C, and HDL-C at baseline and the end of olanzapine therapy. It's important to note that TG levels are susceptible to the quality of the meals slightly before bloodwork.

### Study design

**Figure 1:**
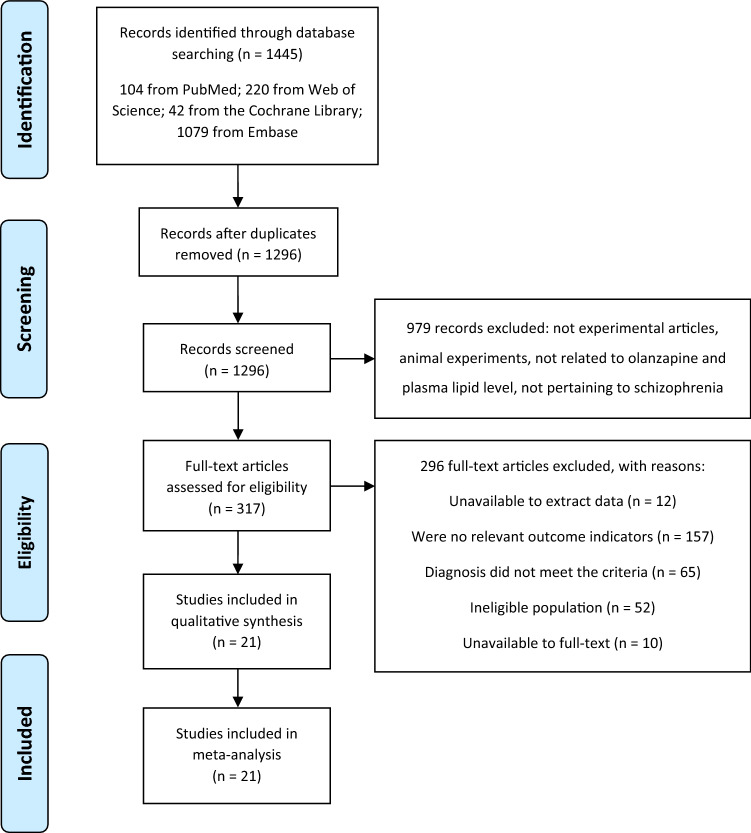
Flowchart of included and excluded reports. This flowchart summarizes the process of document retrieval and selection according to the inclusion criteria.

Randomized controlled clinical trials and cohort studies were included. Furthermore, other non-randomized controlled studies such as self-controlled studies concerning the effects of olanzapine on plasma lipids were also included. Indeed, not only schizophrenia itself may affect the metabolic state including blood lipid metabolism in patients, but the eating habits and exercise in each of individuals is quite different, hence, we conducted a single-arm meta-analysis rather than a pairwise meta-analysis of olanzapine against various control groups. We excluded studies that satisfied one of the following criteria: (1) non-clinical studies, such as animal, cell, genetic and drug metabolism studies; (2) unpublished papers, letters, case reports, reviews, and meta-analyses; (3) the full text not available; (4) the statistical methods were misapplied. A flow chart concerning the process of the identification, inclusion, and exclusion of studies is shown in Fig. 1.

### Data extraction

Data from studies were entered in a pre-specified spreadsheet in Microsoft Excel 2019 and EndNote 8.1. The following details of patients were extracted from each of the included reports: (1) surname name of the first author and the year of publication; (2) institution and country of each study; (3) characteristics about the subjects, including mean age, body mass index (BMI), medication times, sample sizes of the olanzapine and control groups; (4) the plasma levels of TG, TC, LDL-C, and HDL-C at baseline and different time points of olanzapine treatment.

### Quality assessment

The quality of each of the included studies was accessed and scored by two independent reviewers to determine the quality of participant selection, comparability, exposures, and outcomes. The methodological biases of the RCTs were judged according to the Cochrane criteria guidelines, and the researchers were required to make judgments of "yes", "no", and "unclear" for each evaluation item. Recommended by the Agency for Healthcare Research and Quality (AHRQ), the Newcastle–Ottawa Quality (NOS) Assessment Scale, was used to evaluate the quality of cohort studies^15^. We also integrated the methodological index for non-randomized studies (MINORS) in our investigation because of its suitability for evaluating the quality of self-controlled studies^16^. The MINORS is composed of 12 evaluation indices, each of which is awarded scores of 0–2 points: a score of 0 indicates unreported information; a score of 1, insufficiently reported information; and a score of 2, sufficiently reported information. Disagreements were discussed, and adjudication was resolved by a third reviewer.

### Statistical analysis

The data pooled from the selected studies were divided into four groups on the basis of the main serum lipid components (TG, TC, LDL-C and HDL-C), and provided the data source for the primary results. Analysis of the relationship between olanzapine treatment duration and serum lipid changes was achieved from the integrated lipids data of different olanzapine intervention duration. Additionally, a potential source of heterogeneity in serum lipids can be the age of the patient. Indeed, numerous reports have concluded that as people age, their plasma lipid levels tend to also increase. Additionally, epidemiologic studies have shown that a greater proportion of the elderly population is dyslipidemic^17^. Hence, in order to avoid the confounding role of age in our assessment and determine the possible source of heterogeneity, we performed an age subgroup analysis as a function of time.

The effect size of the mean differences of TG, TC, LDL-C, and HDL-C between baseline and endpoint in schizophrenia patients was computed using weighted-mean difference (WMD). A forest plot using α = 0.05 to indicate significance was generated to estimate the WMD and its 95% CI. Heterogeneity of the included studies was tested with the Q-statistic and quantified using *I*^*2*^ = 100% × [(Q – degree of freedom)/Q]. An *I*^2^ of > 50% was considered to reflect significant statistical heterogeneity. The random-effects model using the inverse variance heterogeneity method was adopted with *I*^2^ > 50%^18^.

A sensitivity analysis was performed by excluding one study at a time to explore the sources of heterogeneity. Evidence of publication bias was assessed by a visual funnel plot and Egger’s regression asymmetry test. Subgroup analysis was performed to detect the underlying sources of heterogeneity. All analytic processes were conducted with Stata-SE 15.1 statistical software (STATA Corp, College Station, TX, USA).

## Results

### Search results and study characteristics

Our initial search yielded 1445 studies from the PubMed, Web of Science, The Cochrane Library and Embase databases. Of these studies, 1424 articles were excluded on account of the following: (1) duplicated literature, (2) non-experimental literature, (3) animal experiments, (4) unavailability of the full text, (5) unrelated to olanzapine or plasma lipid levels, (6) unrelated to schizophrenia, and (7) unavailability of the originally relevant data (Fig. 1). All included studies were published in English. Our analysis thus included 21 studies and 1790 schizophrenia patients in the olanzapine arm. We also included the randomized open-label trial by Kusumi et al.^19^ to compare the effect of olanzapine orally disintegrating tablet and oral standard tablet on body weight and lipid metabolism in patients with schizophrenia. Since both arms were treated with olanzapine, all patients were included in this meta-analysis. The main characteristics of these studies are listed in Tables 1 and 2***.***

**Table 1:** Characteristics of the included studies.

Study	Location	Randomization groups		No. of patients randomized	
		Treatment arm	Control arm	Treatment arm	Control arm
Berg, 2005^53^	Europe and America	OLA	ZIP	204–215	196–203
Wu, 2006^21^	China	OLA	CLO/RIS/SUL	24	30/29/29
Kane, 2007^54^	Europe and India	OLA		128	126/374
Saddichha, 2008^55^	India	OLA	ZIP/HAL/HG	35	33/31/51
Kryzhanovskaya, 2009^56^	USA and Russia	OLA		49	15
Bushe, 2010^57^	Unclear	OLA	QUE	151	154
Narula, 2010^58^	India	OLA	OLA + TOP	34	33
McDonnell, 2011^22^	26 countries	OLA	OLA LI	448	265
Kusumi, 2012^19^	Japan	ODT	OST	45	40
Schreiner, 2012^59^	15 countries	OLA	PAL	220	239
Hu, 2013^60^	China	OLA	PAL	23	33
Ou, 2013^8^	China	OLA	ZIP	127	128
Modabbernia, 2014^61^	Iran	OLA	OLA + MEL	18	18
Zhang, 2014^62^	China	OLA	QUE/ARI	50	50/50
Suresh, 2016^63^	India	OLA	RIS	36	35
Chen, 2017^20^	China	OLA	ZIP	19	19
Lin, 2018^38^	China	OLA	HG	23	52
Tanaka, 2008^64^	Japan	OLA	–	28	–
Chiu, 2010^10^	China	OLA	–	33	–
Gilles, 2010^65^	Germany	OLA	–	14	–
Salviato Balbao, 2014^9^	Brazil	OLA	–	30	–

*OLA* olanzapine, *ODT* olanzapine orally disintegrating tablet, *ZIP* ziprasidone, *CLO* clozapine, *RIS* risperidone, *SUL* sulpiride, *PAL* paliperidone, *HAL* haloperidol, *HG* healthy group, *QUE* quetiapine, *TOP* topiramate, *OLA LI* olanzapine long-acting injection, *OST* oral standard tablet, *MEL* melatonin, *ARI* aripiprazole.

**Table 2:** Characteristics of the included studies.

Study	Outcome indicators	BMI of treatment arm	Age	Duration of intervention	Design
Berg, 2005^53^	TG/TC/LDL/HDL	–	40.10 ± 11.60	28 weeks	RCT
Wu, 2006^21^	TG/TC	20.65 ± 0.33	34.20 ± 10.30	8 weeks	RCT
Kane, 2007^54^	TG/TC/LDL/HDL	25.5 ± 5.7	36.30 ± 11.20	6 weeks	RCT
Saddichha, 2008^55^	TG/HDL	–	27.50 ± 5.90	6 weeks	RCT
Kryzhanovskaya, 2009^56^	TG/TC/LDL/HDL	23.5 ± 4.6	16.10 ± 1.30	6 weeks	RCT
Bushe, 2010^57^	TG/LDL/HDL	31.15 ± 7.00	41.67 ± 9.53	24 weeks	RCT
Narula, 2010^58^	TG/TC/LDL/HDL	20.2 ± 3.92	31.00 ± 10.09	12 weeks	RCT
McDonnell, 2011^22^	TG/TC/LDL/HDL	26.80 ± 5.00	38.90 ± 11.30	24 weeks	RCT
Kusumi, 2012^19^	TG/TC/HDL	–	44.30 ± 14.00 and 43.80 ± 12.90	48 weeks	RCT
Schreiner, 2012^59^	TG/HDL	20.20 ± 3.92	37.50 ± 11.40	12, 24 weeks	RCT
Hu, 2013^60^	TG/TC/LDL/HDL	21.54 ± 4.22	28.65 ± 7.14	4, 8 and 12 weeks	RCT
Ou, 2013^8^	TG/TC/LDL/HDL	20.69 ± 2.81	27.68 ± 8.04	6 weeks	RCT
Modabbernia, 2014^61^	TG/TC/LDL/HDL	23.2 ± 3.20	32.8 ± 8.2	4 and 8 weeks	RCT
Zhang, 2014^62^	TG/TC/LDL/HDL	–	41.20 ± 13.30	8 weeks	RCT
Suresh, 2016^63^	TC	–	41.50 ± 9.60	48 weeks	RCT
Chen, 2017^20^	TG/TC/LDL	–	older than 60 years	4, 8 and 12 weeks	RCT
Lin, 2018^38^	TG/TC/LDL/HDL	21.56 ± 2.72	30.17 ± 10.89	4 and 8 weeks	CS
Tanaka, 2008^64^	TG/TC/LDL/HDL	22.20 ± 3.70	59.50 ± 10.00	8 and 16 weeks	SS
Chiu, 2010^10^	TG/TC/LDL/HDL	26.00 ± 3.80	37.60 ± 8.00	2, 4 and 8 weeks	SS
Gilles, 2010^65^	TG/LDL/HDL	24.60 ± 4.40	29.00 ± 8.40	6 weeks	SS
Salviato Balbao, 2014^9^	TG/TC/LDL/HDL	24.40 ± 4.01	27.83 ± 8.34	4, 8, 36 and 48 weeks	SS

*TG* triglyceride, *TC* total cholesterol, *LDL* low-density lipoprotein cholesterol, *HDL* how-density lipoprotein cholesterol, *RCT* randomized controlled trail, *CS* cohort study, *SS* self-control study.

### Methodological quality of the included studies

We used the data extracted from the olanzapine arm (not included control arm) of 16 included RCTs. Our analysis, therefore, was primarily concerned with the bias resulting from the integrity of the lipid data at different time points and selective publication of lipid data inconsistent with the hypothesis. The only cohort study assessed medium quality according to NOS Assessment Scale, and most RCTs (94%) provided complete outcome data instead of selective data (Table 3)***.*** Moreover, four self-controlled studies provided sufficient information (Table 4). Thus, the quality of the included literature was generally high.

**Table 3:** Assessment of quality of RCTs.

Source	Random sequence generation	Allocation concealment		Blinding	Incomplete outcome data		Selective reporting	Description of other bias
Berg, 2005^53^	Unclear	Unclear		Low	Low		Unclear	
Wu, 2006^21^	Unclear	Unclear		High	Low		Low	
Kane, 2007^54^	Low	Low		Low	Low		Low	
Saddichha, 2008^55^	Unclear	Unclear		Low	Low		Low	
Kryzhanovskaya, 2009^56^	Unclear	Unclear		Low	Low		Low	The strict inclusion criteria of this study may limit the generalizability of these results
Bushe, 2010^57^	Low	Low		Low	Low		Low	Blood samples may have been taken in a non-fasting state during the study
Narula, 2010^58^	Unclear	Unclear		Low	Low		Low	The dietary intake of the included patients were different
McDonnell, 2011^22^	Low	Low		Low	Low		Low	
Kusumi, 2012^19^	Low	Low		Low	Low		Low	
Schreiner, 2012^59^	Low	Low		High	Low		Low	
Hu, 2013^60^	Low	Low		High	High		Low	The number of people lost may have an impact on the results
Ou, 2013^8^	Unclear	Unclear		High	Low		Low	
Modabbernia, 2014^61^	Unclear	Unclear		Low	Low		Low	
Zhang, 2014^62^	Low	Unclear		Unclear	Low		Low	Not observing long enough; no nonmetabolism-related side effects were detected
Suresh, 2016^63^	Low	Unclear		Low	Low		Low	
Chen, 2017^20^	Low	Unclear		Low	Low		Low	The sample size is too small

The risk of bias is expressed as high, low and unclear; low = low risk; high = high risk; unclear = unclear risk

**Table 4:** Assessment of quality of non-randomized studies.

Methodological items	Tanaka, 2008^64^	Chiu, 2010^10^	Gilles, 2010^65^		Salviato Balbao, 2014^9^
A clearly stated aim	2	2	2		2
Inclusion of consecutive patients	1	1	1		1
Prospective collection of data	2	2	2		2
Endpoints appropriate to the aim of the study	2	2	1		2
Unbiased assessment of the study endpoint	2	2	2		2
Follow-up period appropriate to the aim of the study	0	1	1		1
Loss to follow up less than 5%	2	0	2		2
Prospective calculation of the study size	0	0	0		0
Total score	11	10	11		12

The items are scored 0 (not reported), 1 (reported but inadequate) or 2 (reported and adequate); the ideal score being 16 for non-comparative studies.

### Primary outcomes

**Figure 2:**
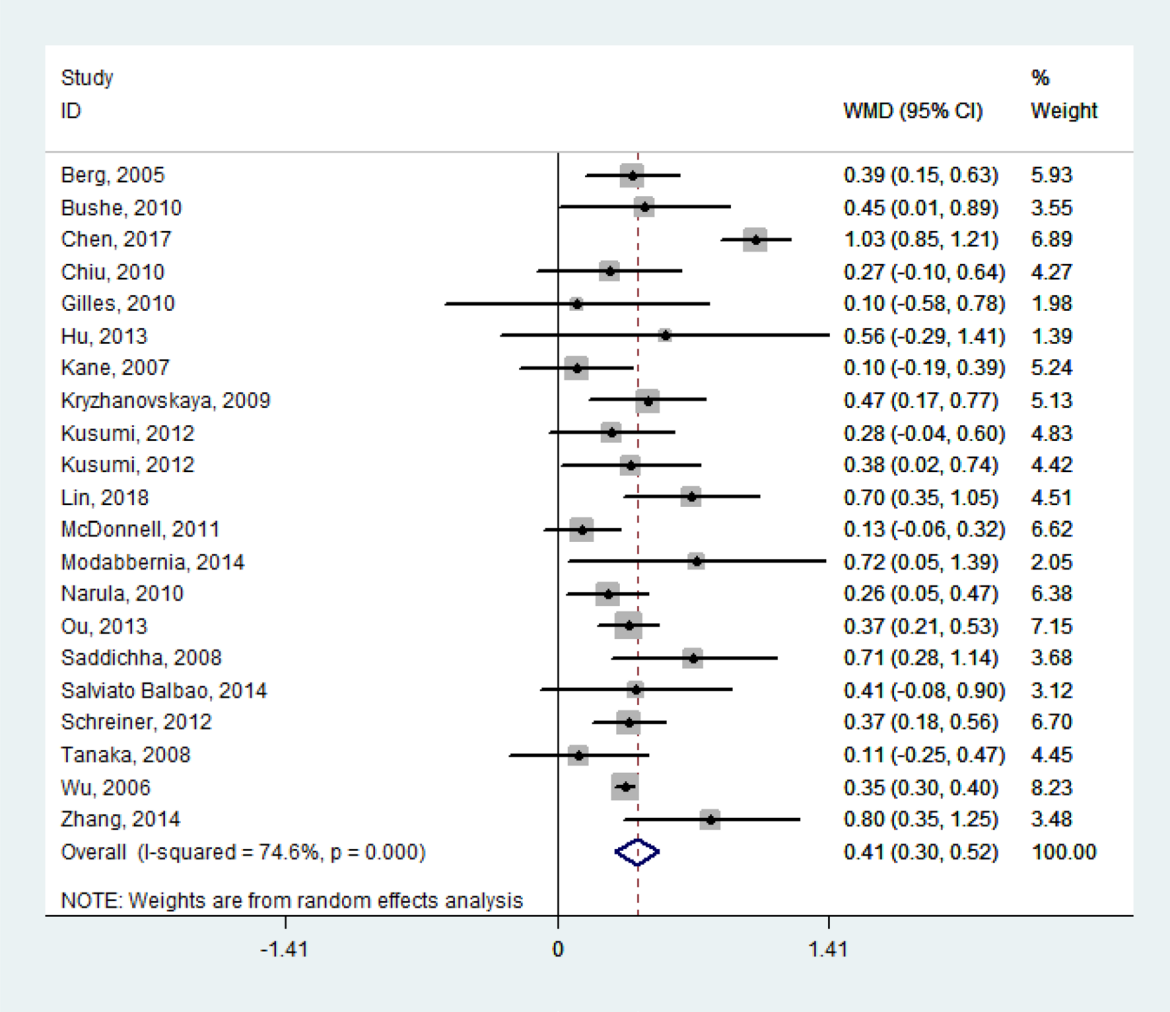
Forest plot and meta-analysis of olanzapine's effects on TG. *WMD* weighted-mean difference; *CI* confidence interval.

**Figure 3:**
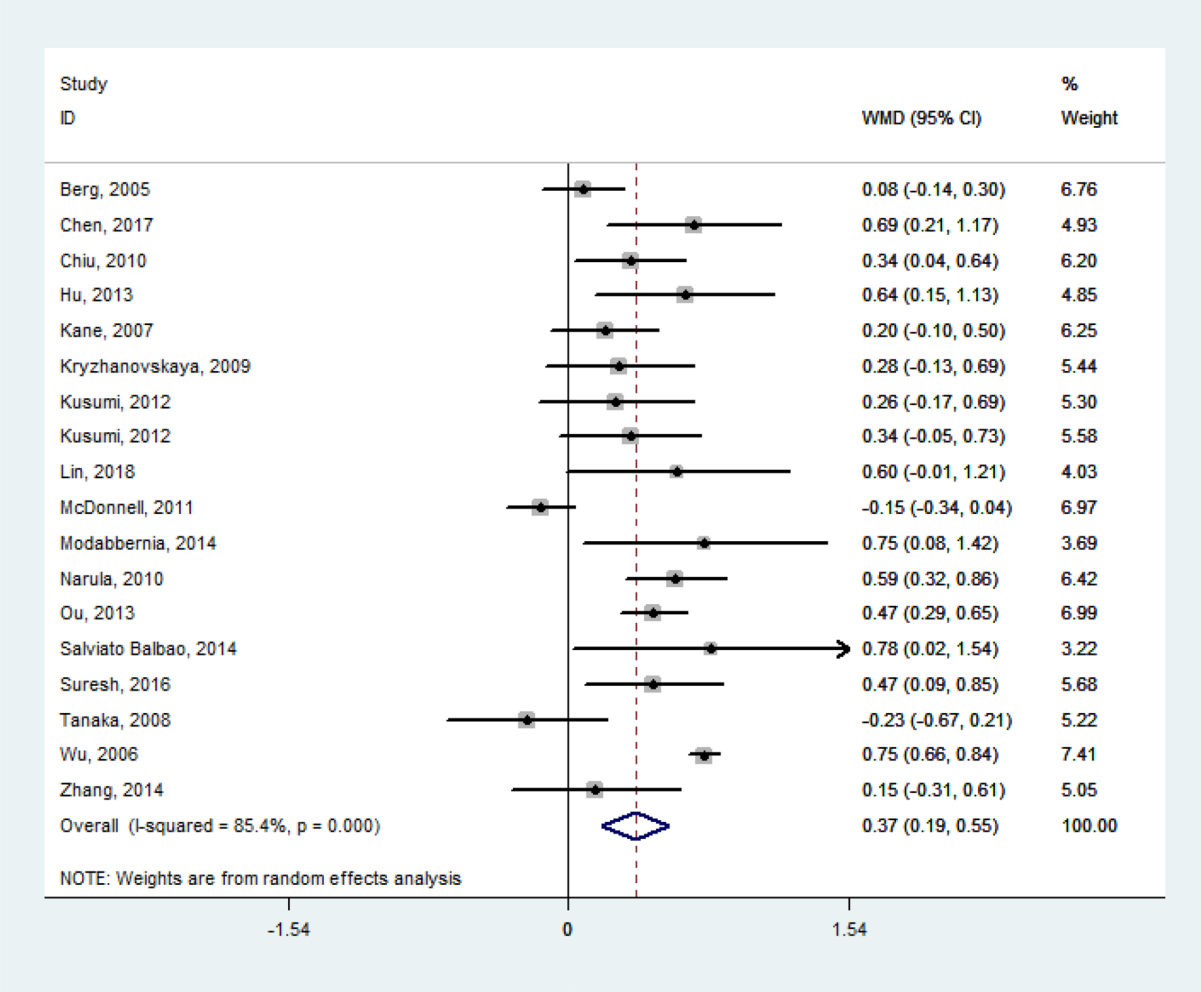
Forest plot and meta-analysis of olanzapine's effects on TC. *WMD* weighted-mean difference; *CI* confidence interval.

**Figure 4:**
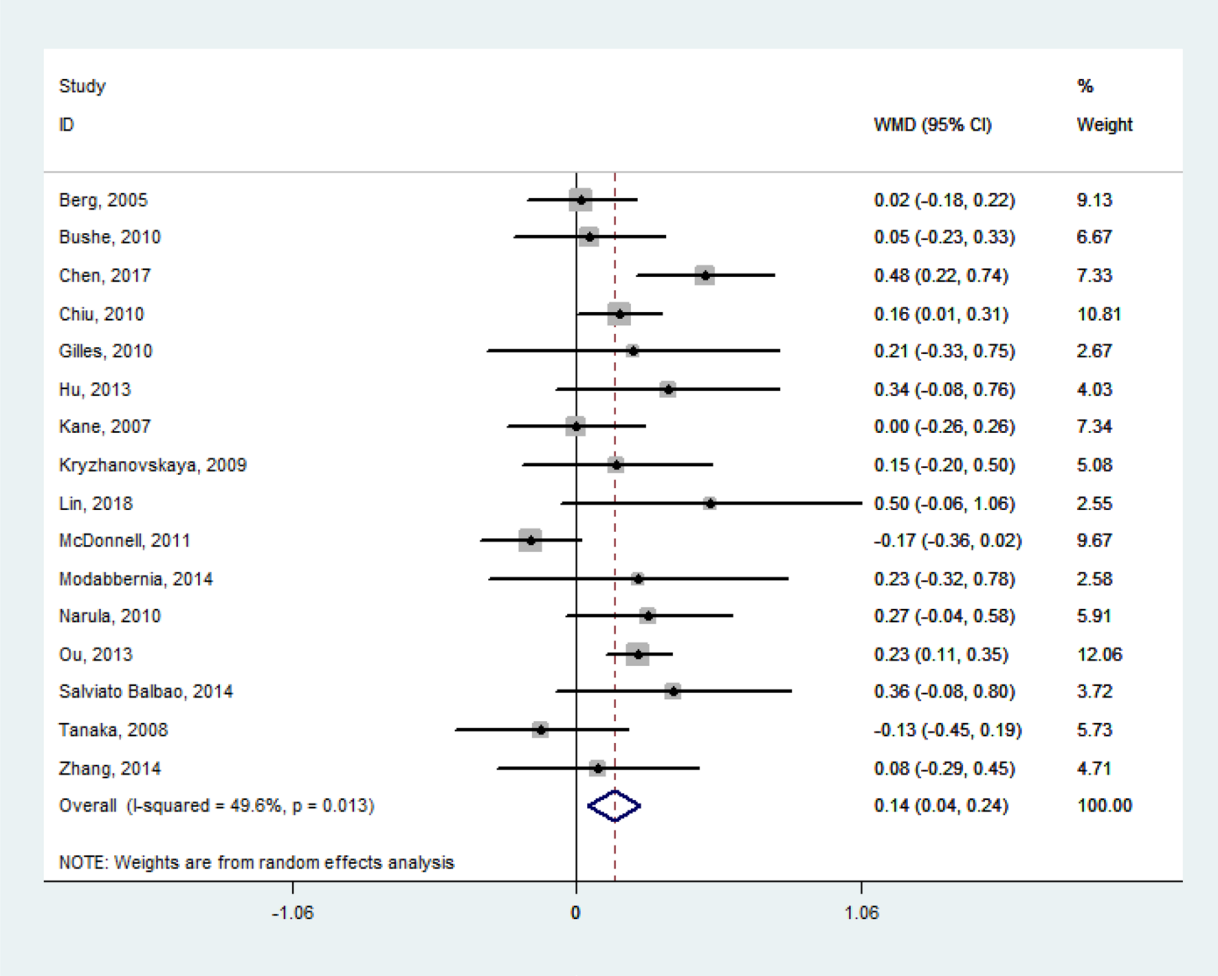
Forest plot and meta-analysis of olanzapine's effects on LDL-C. *WMD* weighted-mean difference; *CI* confidence interval.

**Figure 5:**
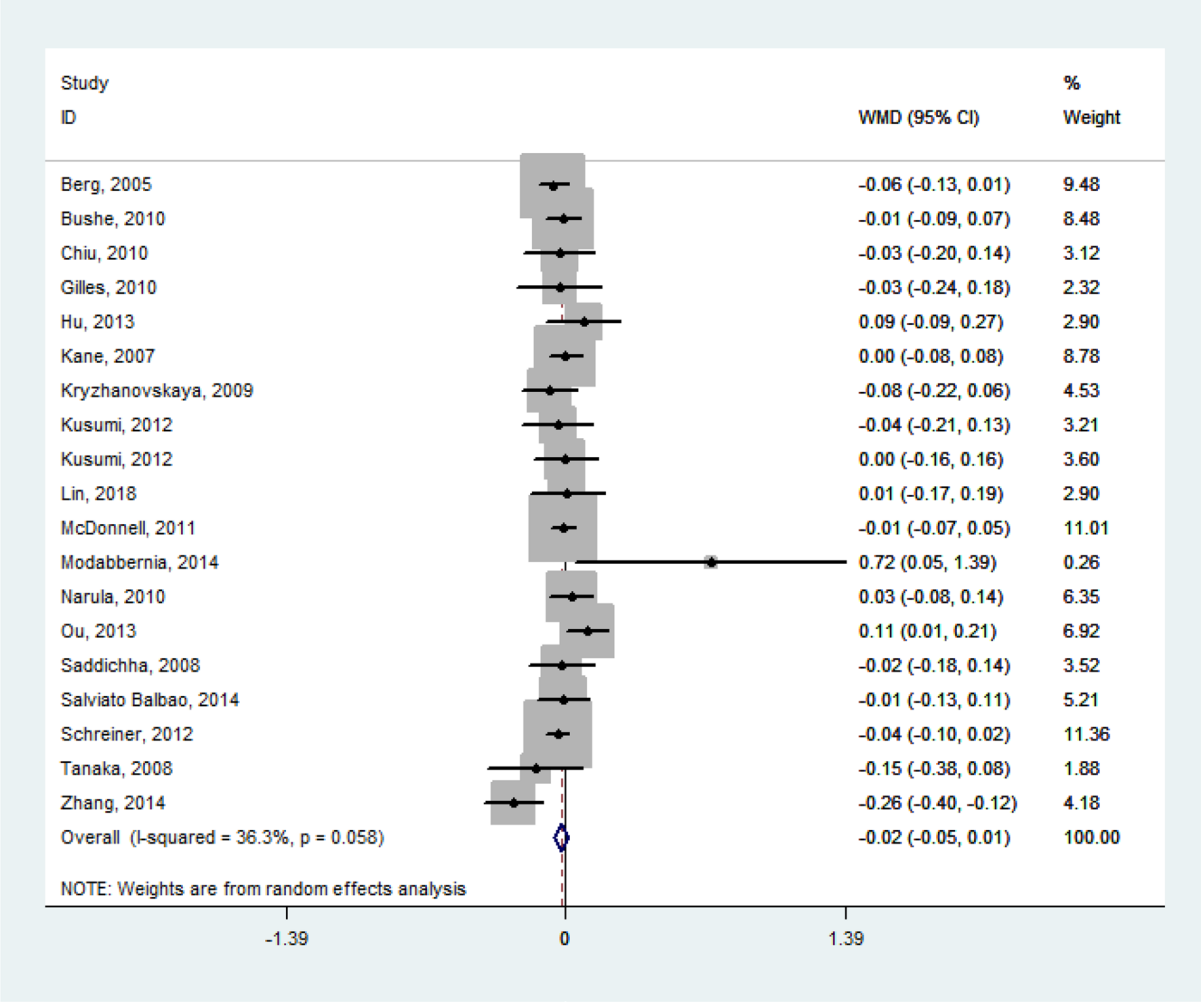
Forest plot and meta-analysis of olanzapine's effects on HDL-C. *WMD* weighted-mean difference; *CI* confidence interval.

The random effect model pooled the WMD of lipid levels from the baseline to the termination of their respective study periods as follows: TG = 0.41 mmol/l (95% confidence interval [CI], 0.30 to 0.52; *P* < 0.001; *I*^*2*^ = 74.6%; *n* = 1569; 21 studies), TC = 0.37 mmol/l (95% CI, 0.19 to 0.55; *P* < 0.001; *I*^*2*^ = 85.4%; *n* = 1185; 18 studies), LDL-C = 0.14 mmol/l (95% CI, 0.04 to 0.24; *P* = 0.005; *I*^*2*^ = 49.6%; *n* = 1159; 16 studies). All the WMD of TG, TC, and LDL-C featured significant heterogeneity. However, no obvious changes were observed in HDL-C (*WMD* = – 0.02 mmol/l; Figs. 2, 3, 4, 5). Taken together, the analysis indicated that olanzapine-induced dyslipidemia is characterized by increases of TG, TC, and LDL-C and no changes in HDL-C.

### Secondary outcomes-changes of lipid profiles depending on the duration of treatment

The effects of olanzapine intervention duration on lipid levels were further analyzed because the accumulation effect of the drug may influence the final detected serum lipid level. The time points analyzed included the following: baseline, 4 weeks, 6 weeks, 8 weeks, 12 weeks, and ≥ 24 weeks (data of ≥ 24 weeks were integrated).

The WMDs of plasma TG changes from baseline to the five end time points were as follows: 4 weeks = 0.57 mmol/l (95% CI, 0.28 to 0.87; *I*^*2*^ = 67.4%), 6 weeks = 0.36 mmol/l (95% CI, 0.18 to 0.54; *I*^*2*^ = 39.7%), 8 weeks = 0.48 mmol/l (95% CI, 0.30 to 0.66; *I*^*2*^ = 67.7%), 12 weeks = 0.55 mmol/l (95% CI, 0.09 to 1.00; *I*^*2*^ = 92.3%), and ≥ 24 weeks = 0.30 mmol/l (95% CI, 0.20 to 0.40; *I*^*2*^ = 0.0%). All WMDs of the above TG changes were significant (*P* < 0.05 for all) (Table 5), showing that olanzapine therapy will increase plasma TG levels in patients with schizophrenia after 4 weeks and that this effect will persist across the remaining period of medication.

**Table 5:** Meta-analysis of secondary outcomes of different intervention times.

Secondary outcomes		Studies, no	Subjects, no	WMD	95% CI	p value	Study heterogeneity			
							X^2^	df	I^2^, %	p value
**TG**	4 weeks	6	146	0.57	0.28, 0.87	0.000	15.36	5	67.40%	0.009
	6 weeks	5	351	0.36	0.18, 0.54	0.000	6.64	4	39.70%	0.000
	8 weeks	10	278	0.48	0.30, 0.66	0.000	27.86	9	67.70%	0.001
	12 weeks	4	296	0.55	0.09, 1.00	0.019	39.05	3	92.30%	0.000
	≥ 24 weeks	7	966	0.30	0.20, 0.40	0.000	4.65	6	0.00%	0.590
**TC**	4 weeks	6	146	0.34	0.10, 0.58	0.006	7.28	5	31.30%	0.201
	6 weeks	3	302	0.36	0.19, 0.54	0.000	2.57	2	22.30%	0.276
	8 weeks	9	248	0.44	0.19, 0.69	0.001	29.78	8	73.10%	0.000
	12 weeks	3	76	0.62	0.41, 0.83	0.000	0.14	2	0.00%	0.935
	≥ 24 weeks	6	631	0.20	− 0.04, 0.44	0.095	14.79	5	66.20%	0.011
**LDL**	4 weeks	6	146	0.14	0.03, 0.25	0.014	5.09	5	1.80%	0.405
	6 weeks	4	312	0.19	0.08, 0.29	0.000	2.58	3	0.00%	0.461
	8 weeks	8	224	0.18	0.07, 0.29	0.001	7.62	7	8.10%	0.368
	12 weeks	3	76	0.39	0.21, 0.56	0.000	1.10	2	0.00%	0.578
	≥ 24 weeks	4	619	− 0.02	− 0.17, 0.13	0.810	4.35	3	31.00%	0.226
**HDL**	4 weeks	5	127	− 0.02	− 0.09, 0.05	0.514	1.32	4	0.00%	0.858
	6 weeks	5	349	0.01	− 0.06, 0.08	0.772	5.84	4	31.50%	0.212
	8 weeks	6	177	− 0.03	− 0.13, 0.07	0.567	12.97	5	61.50%	0.024
	12 weeks	3	277	− 0.00	− 0.05, 0.05	0.952	1.69	2	0.00%	0.429
	≥ 24 weeks	7	957	− 0.03	− 0.06, 0.00	0.069	1.56	6	0.00%	0.955

*WMD* weighted mean difference, *df* degrees of freedom, *CI* confidence interval.

The WMDs of TC changes at 4, 6, 8 and 12 weeks were 0.34 mmol/l (95% CI, 0.10 to 0.58; *I*^*2*^ = 31.3%), 0.36 mmol/l (95% CI, 0.19 to 0.54; *I*^*2*^ = 22.3%), 0.44 mmol/l (95% CI, 0.19 to 0.69; *I*^*2*^ = 73.1%), and 0.62 mmol/l (95% CI, 0.41 to 0.83; *I*^*2*^ = 0.0%), respectively. All WMDs at the four end time points mentioned were significant (*P* < 0.05 for all). Interestingly, no significant changes were found at ≥ 24 weeks (WMD = 0.20 mmol/l; 95% CI = – 0.04 to 0.44; *P* = 0.10; *I*^*2*^ = 66.2%) (Table 5).

For LDL-C, the significant WMDs were obtained at four end time points as follows: 4 weeks = 0.14 mmol/l (95% CI, 0.03 to 0.25; *I*^*2*^ = 1.8%), 6 weeks = 0.19 mmol/l (95% CI, 0.08 to 0.29; *I*^*2*^ = 0.0%), 8 weeks = 0.18 mmol/l (95% CI, 0.07 to 0.29; *I*^*2*^ = 8.1%), and 12 weeks = 0.39 mmol/l (95% CI, 0.21 to 0.56; *I*^*2*^ = 0.0%) (*P* < 0.05 for all). Interestingly, similar to the data concerning TC, no significant LDL-C changes were found at ≥ 24 weeks (*WMD* = – 0.02 mmol/l; 95% CI, – 0.17 to 0.13; *P* = 0.81; *I*^*2*^ = 31.0%) (Table 5).

As shown in Table 5, the WMDs of HDL-C at all end time points were as follows: 4 weeks = – 0.02 mmol/l (95% CI, – 0.09 to 0.05; *I*^*2*^ = 0.0%), 6 weeks = 0.01 mmol/l (95% CI, – 0.06 to 0.08; *I*^*2*^ = 31.5%), 8 weeks = – 0.03 mmol/l (95% CI, – 0.13 to 0.07; *I*^*2*^ = 61.5%), 12 weeks = – 0.00 mmol/l (95% CI, – 0.05 to 0.05; *I*^*2*^ = 0.0%), and ≥ 24 weeks = – 0.03 mmol/l (95% CI, – 0.06 to 0.00; *I*^*2*^ = 0.0%). Of note, no significant changes in HDL-C were found after olanzapine treatment (*P* > 0.05 for all).

### Subgroup analysis according to age

Considering the potential heterogeneity among included studies, an age subgroup analysis was performed (Table 6). The age subgroup analysis was only conducted in groups with an I^*2*^ of ≥ 50%. Meta-analyses could not be conducted in subgroups for which data from only one study was available. The WMD of TG changes from baseline to 12 weeks among patients with schizophrenia (mean age < 40 years) was 0.30 mmol/l (95% CI, 0.16 to 0.45; *I*^*2*^ = 0.0%). For HDL-C, the WMD was 0.02 mmol/l (95% CI, – 0.05 to 0.09; *I*^*2*^ = 0.0%) in patients with schizophrenia (mean age < 40 years) that had undergone an 8-week olanzapine intervention. The groups including TG at 4 weeks, 8 weeks, TC at 8 weeks, and TC ≥ 24 weeks with an I^*2*^ that exceeded 50% after age subgroup analyses required further analysis (see “Sensitivity analysis and potential sources of heterogeneity”).

**Table 6:** Meta-analysis of subgroup according to age.

Subgroup outcomes	Studies, no	Subjects, no	WMD	95% CI	p value	Study heterogeneity			
						X^2^	df	I^2^, %	p value
TG—4 weeks (age < 40)	5	127	0.51	0.16, 0.86	0.004	9.48	4	57.80%	0.050
TG—8 weeks (age < 40)	6	151	0.37	0.29, 0.45	0.000	5.21	5	4.10%	0.390
TG—8 weeks (age ≥ 40)	4	127	0.53	0.12, 0.94	0.012	15.34	3	80.40%	0.002
TG—12 weeks (age < 40)	3	277	0.30	0.16, 0.45	0.000	0.57	2	0.00%	0.751
TC—8 weeks (age < 40)	6	151	0.62	0.43, 0.80	0.000	7.38	5	32.30%	0.194
TC—8 weeks (age ≥ 40)	3	97	0.15	0.09, 1.51	0.516	5.59	2	64.20%	0.061
TC—≥ 24 weeks (age < 40)	2	295	0.19	− 0.62, 0.99	0.653	4.36	1	77.10%	0.037
TC—≥ 24 weeks (age ≥ 40)	4	336	0.27	0.05, 0.42	0.011	3.63	3	17.40%	0.304
HDL—8 weeks (age < 40)	5	127	0.02	− 0.05, 0.09	0.615	1.48	4	0.00%	0.831

Subgroups were divided according to age over 40 years or not.

*WMD* weight mean difference, *df* degrees of freedom, *CI* confidence interval.

Our findings suggest that the age of the patients is an important characteristic in determining the effects of olanzapine on TG and HDL-C. However, we could not analyze the magnitude of the variations in serum lipids and therefore, could not establish a link between the magnitude of the change in serum lipids and the age of the patients from this meta-analysis.

### Sensitivity analysis and potential sources of heterogeneity

A sensitivity analysis was required to identify the sources of heterogeneity in our findings. Our analysis revealed that the studies by Chen et al*.*^20^, Wu et al*.*^21^ and McDonnell et al*.*^22^ were the main causes of heterogeneity (Supplementary Figure S1, S2, S3 and S4) and their exclusion effectively removed or decreased the variations (TG at 4 weeks: *I*^*2*^ = 17.2%; TG at 8 weeks: *I*^*2*^ = 59.4%; TC at 8 weeks: *I*^*2*^ = 35.4%; TC ≥ 24 weeks: *I*^*2*^ = 20.5%). Notably, the stability of our results was substantiated by the consistency of the statistical significance obtained after their exclusion (TG at 4 weeks: *P* < 0.001; TG at 8 weeks: *P* < 0.001; TC at 8 weeks: *P* = 0.002). Finally, the P value of TC ≥ 24 weeks group changed to 0.005 (statistically significant) after the exclusion, revealing that the accuracy of the results in the study of McDonnell et al*.*^22^ was debatable.

### Publication bias

**Figure 6:**
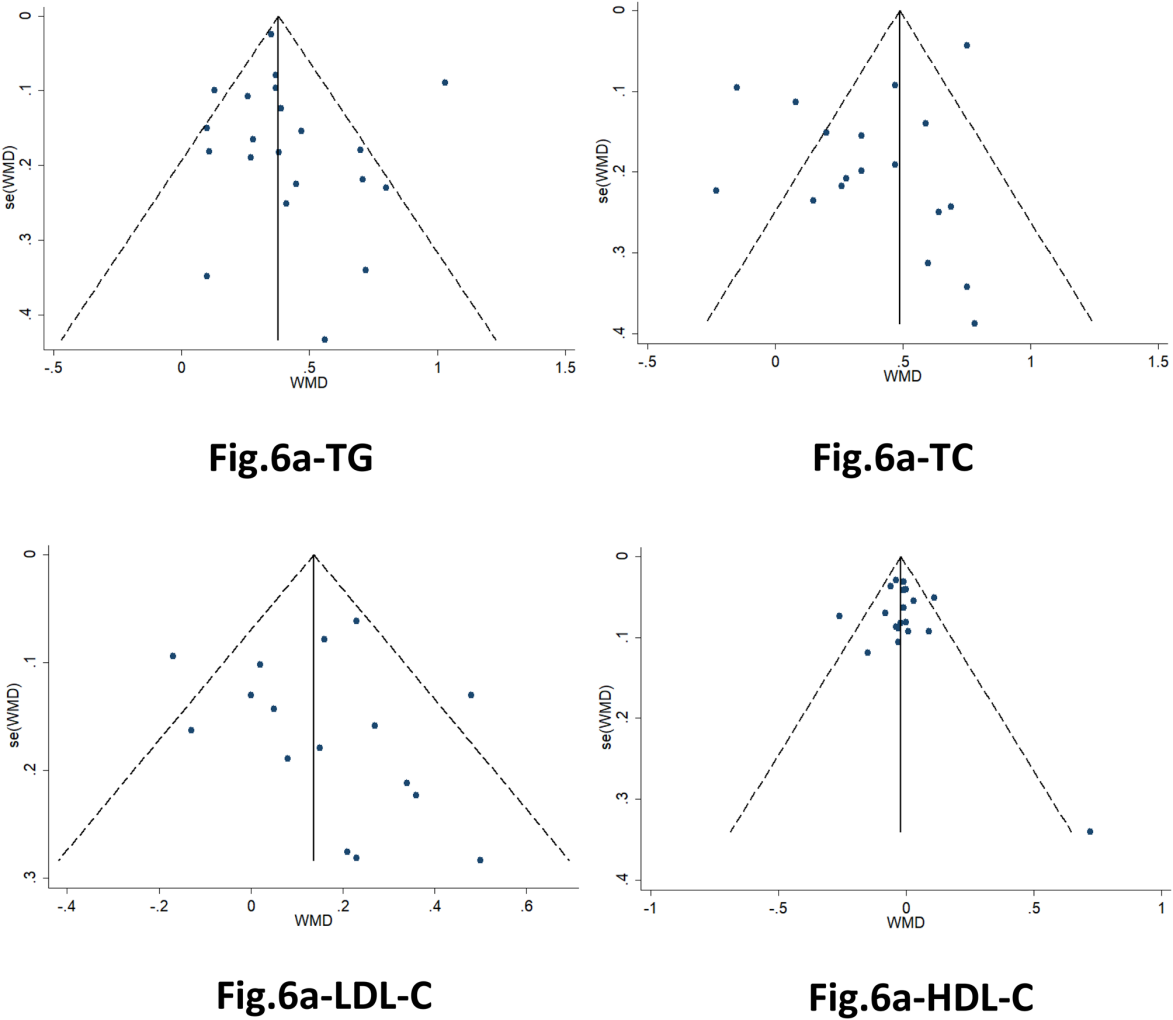
Funnel plots illustrating the publication bias of the four groups of different lipid components. *WMD* weighted mean difference; *se* standard error.

Most studies were located within the 95% CIs of a symmetrical distribution (Fig. 6). In addition, *P* values for the Egger tests were > 0.05, indicating no significant publication bias.

## Discussion

In this current meta-analysis of 21 studies on the effects of olanzapine on lipid profiles in patients with schizophrenia, we demonstrated that the clinical features of dyslipidemia were characterized by an elevation of TG, TC, and LDL-C, with no significant changes in HDL-C. Furthermore, we analyzed the effects of the duration of olanzapine treatment on lipid profiles in patients with schizophrenia, revealed that 4 weeks of olanzapine treatment resulted in abnormal serum lipid levels. Even if the duration of olanzapine-therapy was prolonged, the effect of olanzapine on modulating serum levels of lipid could persist synchronously.

Interestingly, following the exclusion of the findings by McDonnell et al*.*^22^, there was a significant increase in TC level in the TC ≥ 24 weeks group (*P* = 0.005). A review of the research revealed significant decreases in the mean fasting serum TC levels from baseline to end point^22^. The conclusion of McDonnell’s group might be debatable because the long-term diet control was not discussed in the study, and its design included an acute period of stabilization on oral olanzapine before random assignment to therapy^22^. Meanwhile, there were no significant changes in the LDL-C levels after 24-week treatment. We postulate that it might be due to the variations in the sample sizes, ethnicities, and clinical characteristics of the populations in the selected studies. Alternately, the return to baseline levels could have been caused by some form of tolerance to olanzapine. Moreover, the studies were found to have relatively significant heterogeneity (*I*^*2*^ ≥ 50% and *P* < 0.05) in TG, TC and LDL-C but not HDL-C (*I*^*2*^ = 36.3%, *P* = 0.058). The heterogeneity could partly be explained by the different age groups included as a decrease in heterogeneity was observed after age subgroup analyses. Other sources of heterogeneity could be the discordant measurements and results between different studies, resulting in impaired validity of the conclusions. Nevertheless, no publication bias of the literature was detected, further suggesting that the heterogeneity did not result from publication bias. To the best of our knowledge, previous meta-analyses mostly focused on lipid comparison between different antipsychotics, this is the first meta-analysis to investigate the correlation of olanzapine and dyslipidemia in patients with schizophrenia, especially the effect of olanzapine intervention duration on lipid metabolism. Our findings could contribute to the monitoring and management of dyslipidemia in patients with schizophrenia undergoing olanzapine treatment.

Currently, growing evidence suggests that olanzapine treatment can promote several cardio-metabolic disorders such as dyslipidemia in schizophrenic patients, contributing to markedly shorter longevities as compared to the general population^23^. In the first published cases of olanzapine-associated hypertriglyceridemia, Sheitman et al*.*^24^ followed nine patients on olanzapine for an average of 16 months and observed an increase in serum TG from the baseline mean of 170 mg/dl (range 25–200 mg/dl) to the mean of 240 mg/dl (range 135–369 mg/dl), with five patients sustaining > 50% increase in serum TG. Further research associated olanzapine with a roughly five-time increased risk of developing hyperlipidemia as compared with no antipsychotic exposure^25^. As the role of dyslipidemia in CVD development has been well-documented, the increased risk of CVD attributed to olanzapine-induced dyslipidemia is likewise clear^12^: with each 1 mmol/l increase in TG, the risk of CVD increases by ≈ 12% and 37% in men and women, respectively^26^. Furthermore, a meta-analysis of 90,056 individuals in 14 randomized trials showed that each 1 mmol/L drop of LDL-C reduces the risk of CVD by 23%^27^. The WMD of lipid levels from the baseline to endpoint were 0.41 mmol/l of TG, 0.37 mmol/l of TC, and 0.14 mmol/l of LDL-C, respectively. These figures stress the importance of managing olanzapine-induced dyslipidemia especially TG and LDL-C in patients with schizophrenia to the prevention of CVD in this population^28^. Besides, the fibrates and the statins are recommended to effectively control the TG and LDL-C levels, which may be also used to treat olanzapine-associated hypertriglyceridemia and reduce the plasma level of LDL-C. Therefore, it has been well-established that olanzapine treatment—specifically, olanzapine-induced dyslipidemia—contributes to high cardiovascular risk among patients with schizophrenia^7,29^.

Notably, regular lipid monitoring and physical health care for schizophrenic patients are usually suboptimal and may contribute to the high prevalence of CVD and increased mortality^30,31^. However, to date, the clinical features of olanzapine-induced dyslipidemia have remained controversial because of inconsistent data between studies^7–11^. In an early study, Osser et al*.* observed an elevation of plasma TG levels after olanzapine treatment^32^. The CATIE Schizophrenia Trial observed higher TG levels in patients with schizophrenia who were treated with olanzapine for 3 months than in those treated with ziprasidone^33^. This finding is supported by two recent studies that have also demonstrated an olanzapine-induced elevation of TG in patients with schizophrenia^34,35^. However, the data about the effects of olanzapine on plasma cholesterol (TC, HDL-C and LDL-C) levels were inconsistent. A meta-analysis in 2010 observed an increase of TC after olanzapine treatment^36^, but no significant TC elevation was shown in another meta-analysis of 18 studies^37^. Additionally, the data obtained from Chinese populations of individuals with schizophrenia also remain controversial. The studies by Lin et al*.*^38^ and Chiu et al*.*^10^ revealed that olanzapine increased TC and LDL-C in this population, with no obvious changes in HDL-C^10,38^. By contrast, no effects of olanzapine on TC and LDL-C were found by Huang et al*.*^7^. Concerning HDL-C, Ou et al. observed an increase after olanzapine therapy^8^, whereas an olanzapine-induced reduction of HDL-C was detected in another study^11^. Our meta-analysis demonstrates that olanzapine-induced dyslipidemia is characterized by elevations of TG, TC, and LDL-C, with no significant changes in HDL-C.

Our study also characterized the influence of olanzapine on serum lipids after 4 weeks of treatment and may be beneficial clinically to monitor the plasma lipid levels in a timely manner and help guide appropriate interventions if necessary. Of note, two studies concluded that TG levels increased significantly after 2–3 weeks of olanzapine treatment and found the increase in TG levels to occur before the weight gain and other lipid levels changes^7,39^. However, their relatively small sample sizes of 13 and 15 patients, make it difficult to generalize their findings to a broader clinical population of patients on olanzapine. More extensive studies will be required to validate the findings.

While the mechanism of olanzapine-induced dyslipidemia remains unknown, several factors have been implicated. First, olanzapine antagonizes the 5-hydroxytryptamine and histamine H1 receptors in the hypothalamus^40,41^, and promotes adenosine monophosphate activated protein kinase (AMPK) phosphorylation in the central nervous system^42^. This in turn results in increased food intake, dyslipidemia and obesity^43^. Dyslipidemia may be a secondary reaction to olanzapine-induced weight gain or obesity. Our study only revealed changes in four lipid parameters and did not explore the relationship between lipid profile and body weight. More studies such as meta-analyses or large-scale clinical trials could be conducted to discuss this issue in the future. Besides, the concentrations of circulating leptin and ghrelin were also significantly increased in schizophrenic patients on olanzapine and correlated with increased food intake^44,45^. Second, olanzapine-induced insulin resistance inhibits the activity of lipoprotein lipase, thereby slowing the catabolism of LDL and increasing plasma LDL-C levels^46,47^. In addition, insulin resistance stimulates sterol regulatory element binding protein-1c (SREBP-1c), which enhances the production of very low density lipoprotein in the liver and consequently increases plasma TG levels^48,49^. Third, the molecular mechanisms underlying olanzapine-associated lipid dysregulation are partly understood. Olanzapine enhances lipogenesis directly in the liver by regulating the expression of AMPK, SREBP-1c or peroxisome proliferation-activated receptor in the liver and disturbing the transcription of genes regulating lipid metabolism^50–52^.

Of note, this meta-analysis is subject to several limitations. First, the variance in the reagents and instruments for lipid measurement used among the included studies contributed to heterogeneity. Second, the inclusion of several studies with small samples might impair the authority of our conclusions. Third, this manuscript was marred by currently inefficient resource sharing.

## Conclusion

The present analysis demonstrated that olanzapine-induced dyslipidemia in patients with schizophrenia is characterized by elevated plasma TG, TC, and LDL-C levels, with no significant changes in HDL-C. Dyslipidemia always occurs already by 4 weeks of treatment and, to some extent, exhibits a time-dependent effect. Hence, there is an urgent need for physicians to manage olanzapine-induced dyslipidemia in patients with schizophrenia to prevent the development of CVD in this population. However, a more comprehensive meta-analysis, with unlimited access to the literature, will be required to validate our findings. In addition, further long-term studies that allow the use of lipid-lowering medications upon the emergence of hyperlipidemia will be needed.

## Supplementary information

Supplementary Information.
