# Supplementary material for: Effects of olanzapine treatment on lipid profiles in patients with schizophrenia: a systematic review and meta-analysis
Source: Sci Rep. 2020 Oct 12;10:17028. doi: 10.1038/s41598-020-73983-4 (PMC7552389; doi:10.1038/s41598-020-73983-4)
Supplement: Supplementary file 1 — Supplementary Information. [file 41598_2020_73983_MOESM1_ESM.docx]

**Eﬀects of olanzapine treatment on lipid profiles in patients with schizophrenia: A systematic review and meta-analysis**

Rong Li^1#^, Yiqi Zhang^2^, Wenqiang Zhu^2^, Chen Ding^2^, Wenjie Dai^3^, Xin Su^2^, Wen Dai^2^, Jingmei Xiao^4^, Zhenhua Xing^5^, Xiansheng Huang^2*^

1. Department of Stomatology, The Second Xiangya Hospital, Central South University, Changsha, Hunan, China.

2. Department of Cardiovascular Medicine, The Second Xiangya Hospital, Central South University, Changsha, Hunan, China.

3. Department of Epidemiology and Health Statistics, Xiangya School of Public Health, Central South University, Changsha, Hunan, China.

4. Department of Mental Health, The Second Xiangya Hospital, Central South University, Changsha, Hunan, China.

5. Department of Emergency Medicine, The Second Xiangya Hospital, Central South University, Changsha, Hunan, China.

^#^ First Author

^*^Corresponding Author: Xiansheng Huang ([huangxiansheng@csu.edu.cn](mailto:huangxiansheng@csu.deu.cn))

**Supplementary Table S1.** PRISMA Checklist

| **Section/topic** | **#** | **Checklist item** | **Reported on page #** |
| --- | --- | --- | --- |
| **TITLE** | | |  |
| Title | 1 | Identify the report as a systematic review, meta-analysis, or both. | Page 1 |
| **ABSTRACT** | | |  |
| Structured summary | 2 | Provide a structured summary including, as applicable: background; objectives; data sources; study eligibility criteria, participants, and interventions; study appraisal and synthesis methods; results; limitations; conclusions and implications of key findings; systematic review registration number. | Page 2 |
| **INTRODUCTION** | | |  |
| Rationale | 3 | Describe the rationale for the review in the context of what is already known. | Page 3 to Page 4 |
| Objectives | 4 | Provide an explicit statement of questions being addressed with reference to participants, interventions, comparisons, outcomes, and study design (PICOS). | Page 4 to Page 6 |
| **METHODS** | | |  |
| Protocol and registration | 5 | Indicate if a review protocol exists, if and where it can be accessed (e.g., Web address), and, if available, provide registration information including registration number. | Page 4 |
| Eligibility criteria | 6 | Specify study characteristics (e.g., PICOS, length of follow-up) and report characteristics (e.g., years considered, language, publication status) used as criteria for eligibility, giving rationale. | Page 5 to Page 6 |
| Information sources | 7 | Describe all information sources (e.g., databases with dates of coverage, contact with study authors to identify additional studies) in the search and date last searched. | Page 4 to Page 5 |
| Search | 8 | Present full electronic search strategy for at least one database, including any limits used, such that it could be repeated. | Page 4 to Page 5 |
| Study selection | 9 | State the process for selecting studies (i.e., screening, eligibility, included in systematic review, and, if applicable, included in the meta-analysis). | Page 6 to Page 7 |
| Data collection process | 10 | Describe method of data extraction from reports (e.g., piloted forms, independently, in duplicate) and any processes for obtaining and confirming data from investigators. | Page 7 to Page 8 |
| Data items | 11 | List and define all variables for which data were sought (e.g., PICOS, funding sources) and any assumptions and simplifications made. | Page 7 to Page 8 |
| Risk of bias in individual studies | 12 | Describe methods used for assessing risk of bias of individual studies (including specification of whether this was done at the study or outcome level), and how this information is to be used in any data synthesis. | Page 6 to Page 7 |
| Summary measures | 13 | State the principal summary measures (e.g., risk ratio, difference in means). | Page 7 to Page 8 |
| Synthesis of results | 14 | Describe the methods of handling data and combining results of studies, if done, including measures of consistency (e.g., I^2^) for each meta-analysis. | Page 7 to Page 8 |
| Risk of bias across studies | 15 | Specify any assessment of risk of bias that may affect the cumulative evidence (e.g., publication bias, selective reporting within studies). | Page 6 to Page 7 |
| Additional analyses | 16 | Describe methods of additional analyses (e.g., sensitivity or subgroup analyses, meta-regression), if done, indicating which were pre-specified. | Page 7 to Page 8 |
| **RESULTS** | | |  |
| Study selection | 17 | Give numbers of studies screened, assessed for eligibility, and included in the review, with reasons for exclusions at each stage, ideally with a flow diagram. | Page 8 and Page 9 |
| Study characteristics | 18 | For each study, present characteristics for which data were extracted (e.g., study size, PICOS, follow-up period) and provide the citations. | Page 8 and Page 9 |
| Risk of bias within studies | 19 | Present data on risk of bias of each study and, if available, any outcome level assessment (see item 12). | Page 9 |
| Results of individual studies | 20 | For all outcomes considered (benefits or harms), present, for each study: (a) simple summary data for each intervention group (b) effect estimates and confidence intervals, ideally with a forest plot. | Page 9 to Page 11 |
| Synthesis of results | 21 | Present results of each meta-analysis done, including confidence intervals and measures of consistency. | Page 9 to Page 12 |
| Risk of bias across studies | 22 | Present results of any assessment of risk of bias across studies (see Item 15). | Page 9 |
| Additional analysis | 23 | Give results of additional analyses, if done (e.g., sensitivity or subgroup analyses, meta-regression [see Item 16]). | Page 12 to Page 13 |
| **DISCUSSION** | | |  |
| Summary of evidence | 24 | Summarize the main findings including the strength of evidence for each main outcome; consider their relevance to key groups (e.g., healthcare providers, users, and policy makers). | Page 13 to Page 18 |
| Limitations | 25 | Discuss limitations at study and outcome level (e.g., risk of bias), and at review-level (e.g., incomplete retrieval of identified research, reporting bias). | Page 18 |
| Conclusions | 26 | Provide a general interpretation of the results in the context of other evidence, and implications for future research. | Page 18 to Page 19 |
| **FUNDING** | | |  |
| Funding | 27 | Describe sources of funding for the systematic review and other support (e.g., supply of data); role of funders for the systematic review. | Page 19 |

**
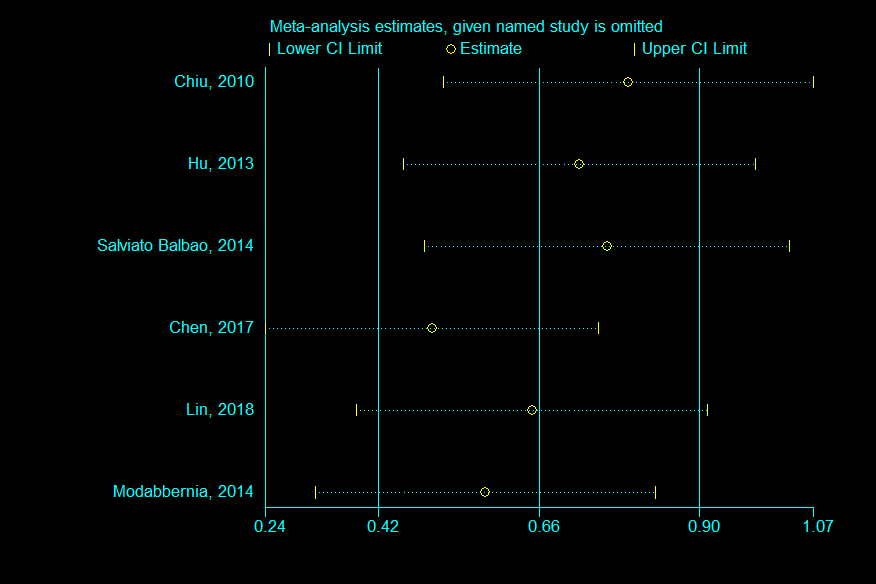
**

**Supplementary Figure S1.** Sensitivity analyses in the group of serum TG level at 4 weeks.

**
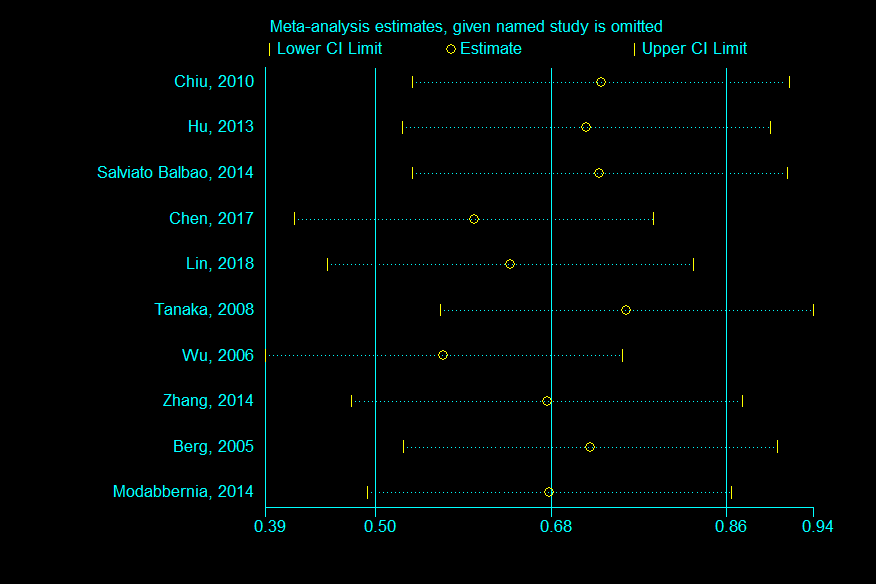
**

**Supplementary Figure S2.** Sensitivity analyses in the group of serum TG level at 8 weeks.


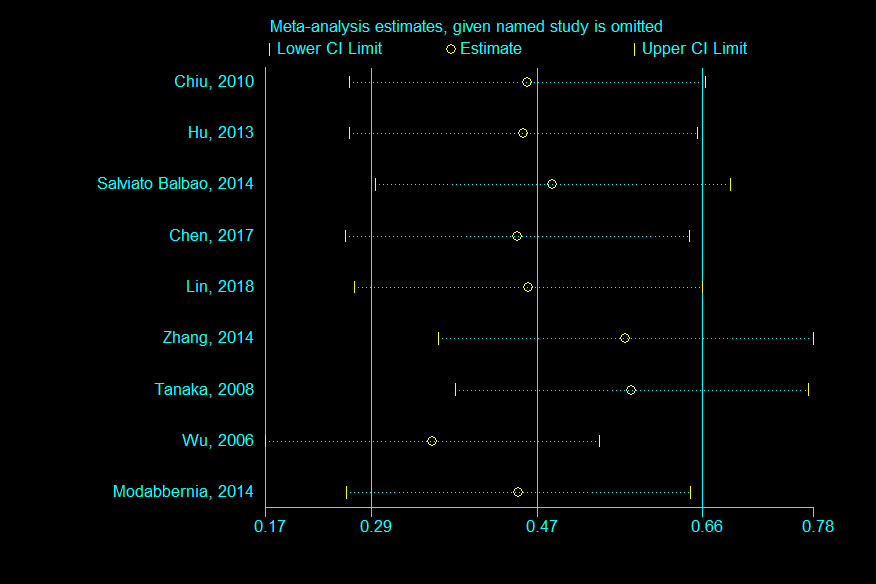


**Supplementary Figure S3.** Sensitivity analyses in the group of serum TC level at 8 weeks.


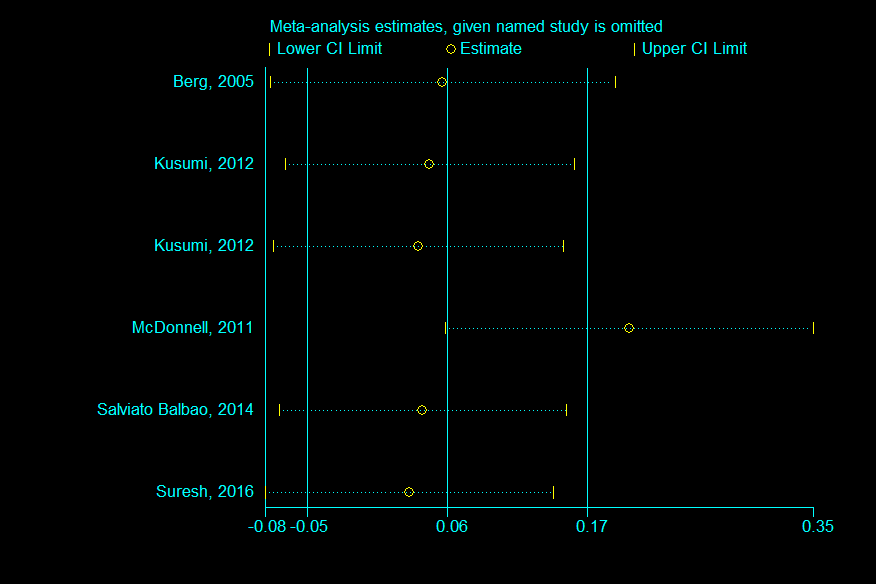


**Supplementary Figure S4.** Sensitivity analyses in the group of serum TC level at 24 weeks and above.
